# Supplementary material for: Meta-analysis of Liver and Heart Transcriptomic Data for Functional Annotation Transfer in Mammalian Orthologs
Source: Comput Struct Biotechnol J. 2017 Aug 26;15:425–32. doi: 10.1016/j.csbj.2017.08.002 (PMC5691612; doi:10.1016/j.csbj.2017.08.002)
Supplement: Supplementary file 6 — Supplementary Figures. [file mmc6.pdf]

Supplementary table caption

Supplementary table 1: GEO series of experiment used in this analysis.

Supplementary table 2: Gene list enrichment analysis of probe cluster, using the full set of probes as a background, identifies ontologies and pathway involved in liver metabolism. FE: Fold Enrichment. BP: Biological process.

Supplementary table 3: SCHype clusters can help identify functionally equivalent orthologs in complex homology families from liver transcriptomic data. homology_group: Homologen homology group id. tax_ID: taxonomic id. gene_ID: entrez gene id. SCHype_cluster_XX columns: are the genes found in the XX SCHype cluster (build using a correlation threshold of 0.5) HT SCHype cluster XX: are the genes found in the XX high threshold SCHype cluster (build using a correlation threshold of 0.75)

Supplementary table 4: Gene list enrichment analysis of heart variable probes, using the full set of probes as a background, identifies ontologies and pathway involved in heart metabolism. FE: Fold Enrichment. BP: Biological process.

Supplementary table 5: SCHype clusters can help identify functionally equivalent orthologs in complex homology families from heart transcriptomic data. homology_group: Homologen homology group id. tax_ID: taxonomic id. gene_ID: entrez gene id. SCHype_cluster_XX columns: are the genes found in the XX SCHype cluster (build using a correlation threshold of 0.5) HT SCHype cluster XX: are the genes found in the XX high threshold SCHype cluster (build using a correlation threshold of 0.75)

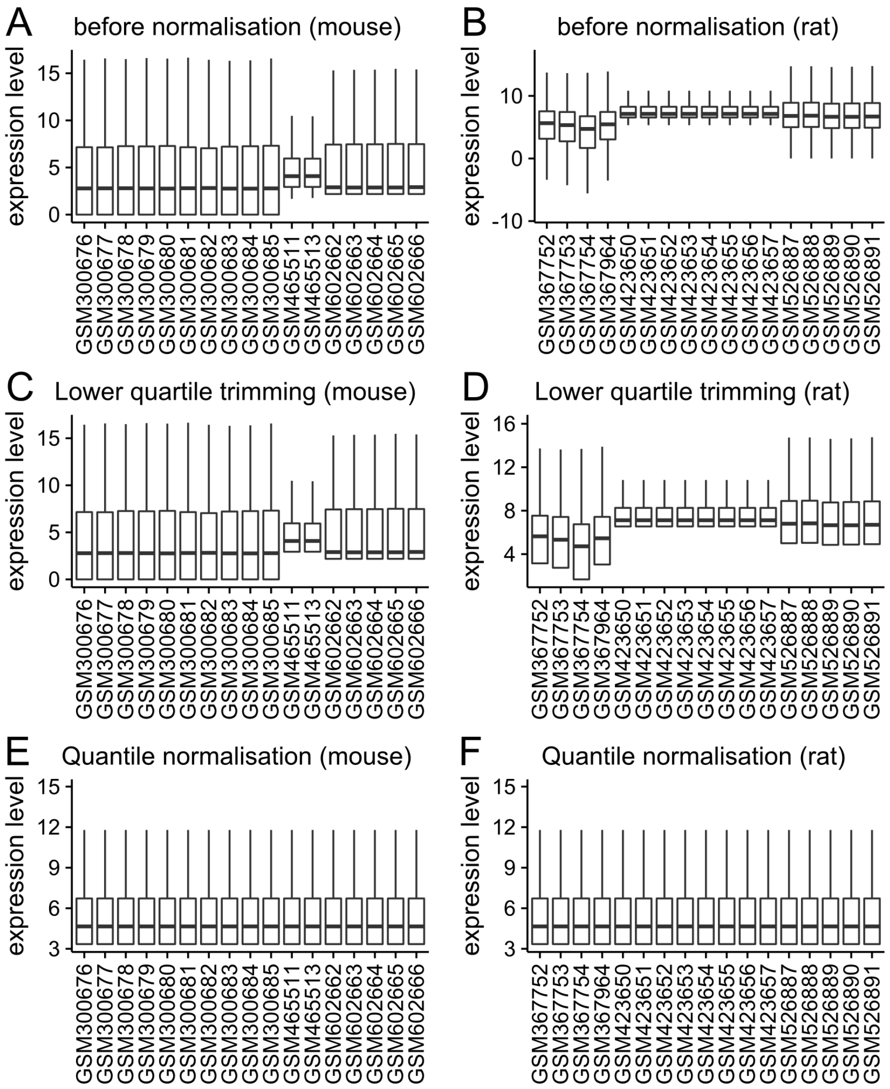

Supplementary figure 1: Normalisation of liver micro-array data. A and B: Before normalisation, micro-array data is heterogeneous, variable from series to series of experiments. C and D: some datasets had a trimmed lower quantile. We thus applied a lower quartile trimming to all samples. E and F: Quantile normalisation was then applied. To obtain similar range of expression values for both species, we used mouse value distribution for rat quantile normalisation (see Methods). A, C, and E: A few representative experiments from mouse. B, F and F: A few representative experiments from rat.

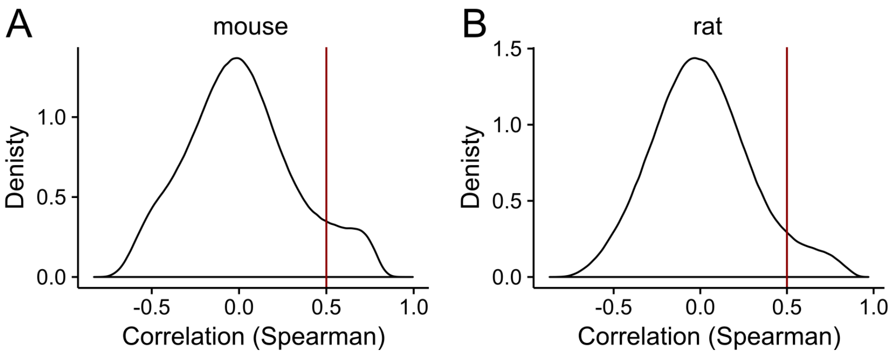

Supplementary figure 2: Distribution or Spearman probe to probe correlation values in mouse (A) and rat (B). Red line indicate the threshold used (0.5) to create the probe graphs used by SCHype.

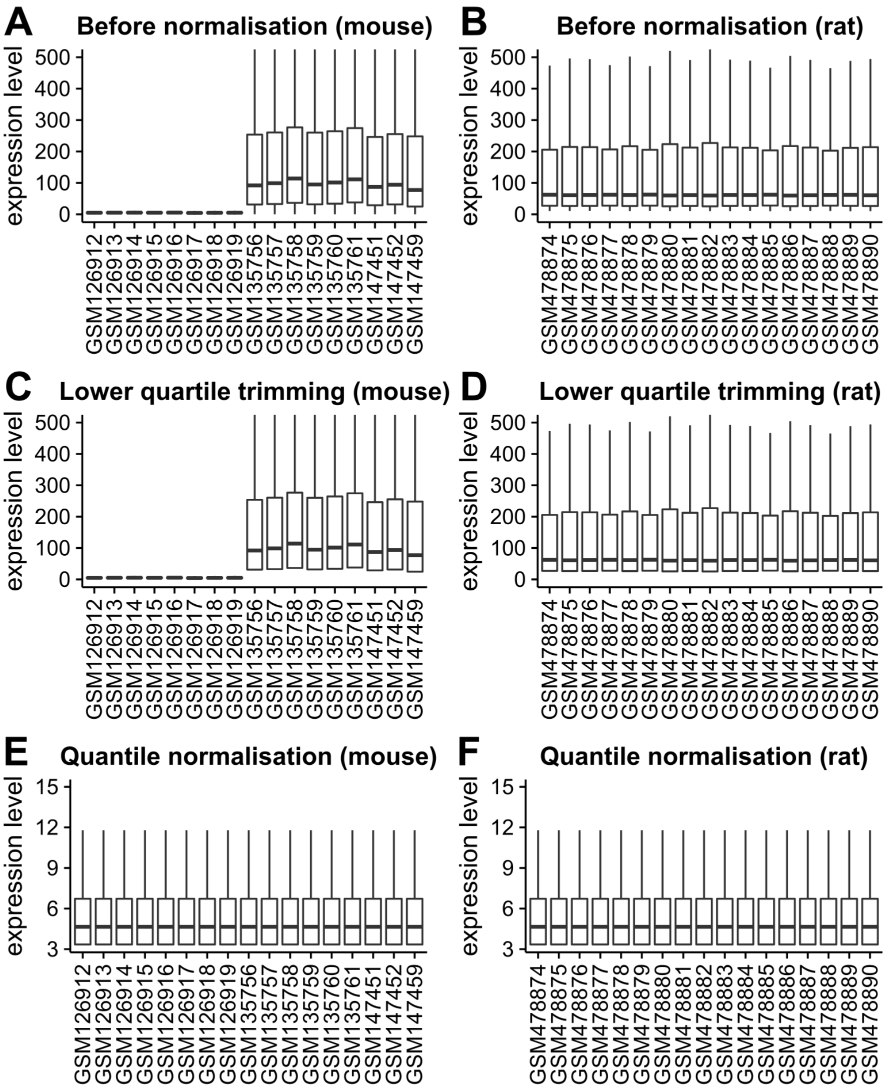

Supplementary figure 3: Normalisation of heart micro-array data. A and B: Before normalisation, micro-array data is heterogeneous, variable from series to series of experiments. C and D: some datasets had a trimmed lower quantile. We thus applied a lower quartile trimming to all samples. E and F: Quantile normalisation was then applied. We used mouse liver value distribution for mouse and rat heart quantile normalisation (see Methods). A, C, and E: A few representative experiments from mouse. B, F and F: A few representative experiments from rat.

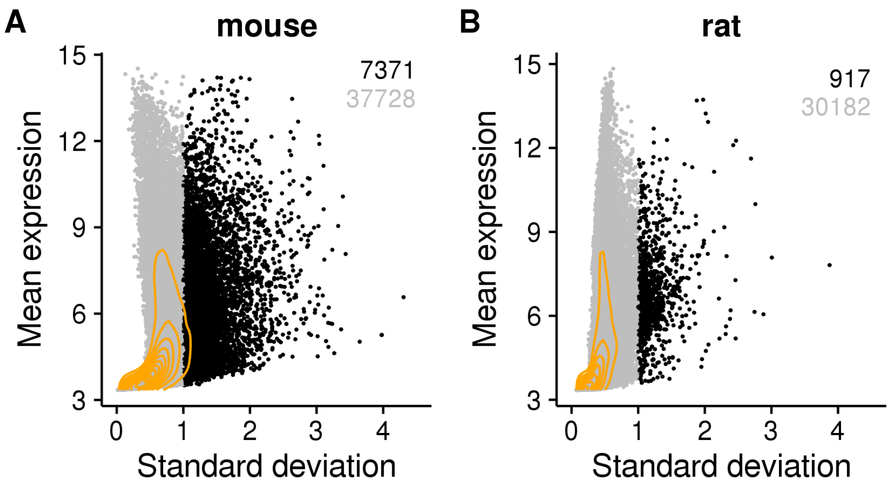

Supplementary figure 4: Identification of variable probes in mouse (A) and rat (B) heart datasets. Each dot represents a single probe. X axis: standard deviation across experiments. Y-axis: mean expression values across experiments (in arbitrary units). In black the probes with a standard deviation ≥ 1, in grey the probes with a standard deviation < 1. Orange lines: 2D kernel density.

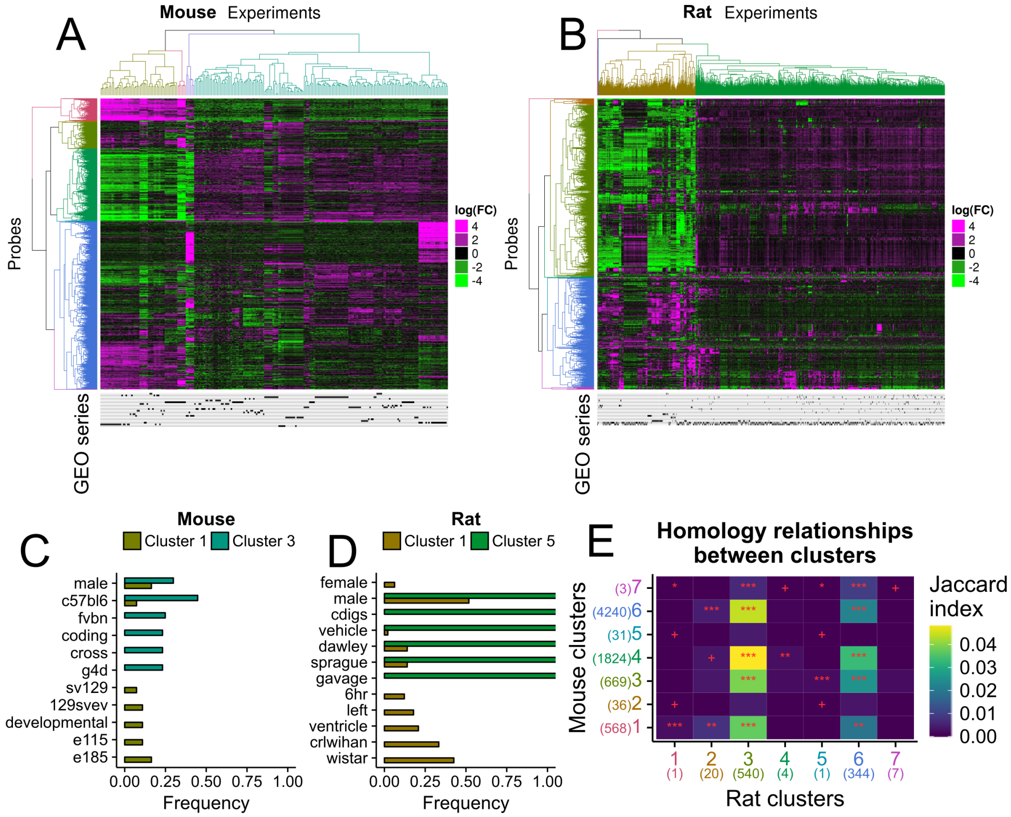

Supplementary figure 5: Hierarchical clustering of variable probes in mouse (A) and in rat (B) heart datasets. Four (mouse) and five (rat) clusters were defined for experiments, and seven for probes, as identified by the dendrogram colours. Below the heatmaps, localisation of experiments from each series are shown in black, one line per series. FC: fold change. C and D. Metadata term frequencies of the two biggest experiment clusters were compared for mouse (C) and rat (D). Colour-code matches the experiments trees in panels A and B. E. Homology relationships between probe clusters between rat (x-axis) and mouse (y-axis). Cell colour: jaccard index. Cell label: Bonferonni adjusted p-values: *** ≤ 0.0001, ** ≤ 0.001, * ≤ 0.01, + ≤ 0.05. Cluster number colours match the probes dendrogram colours in panels A and B. The numbers in parenthesis denote the number of probes in each cluster.

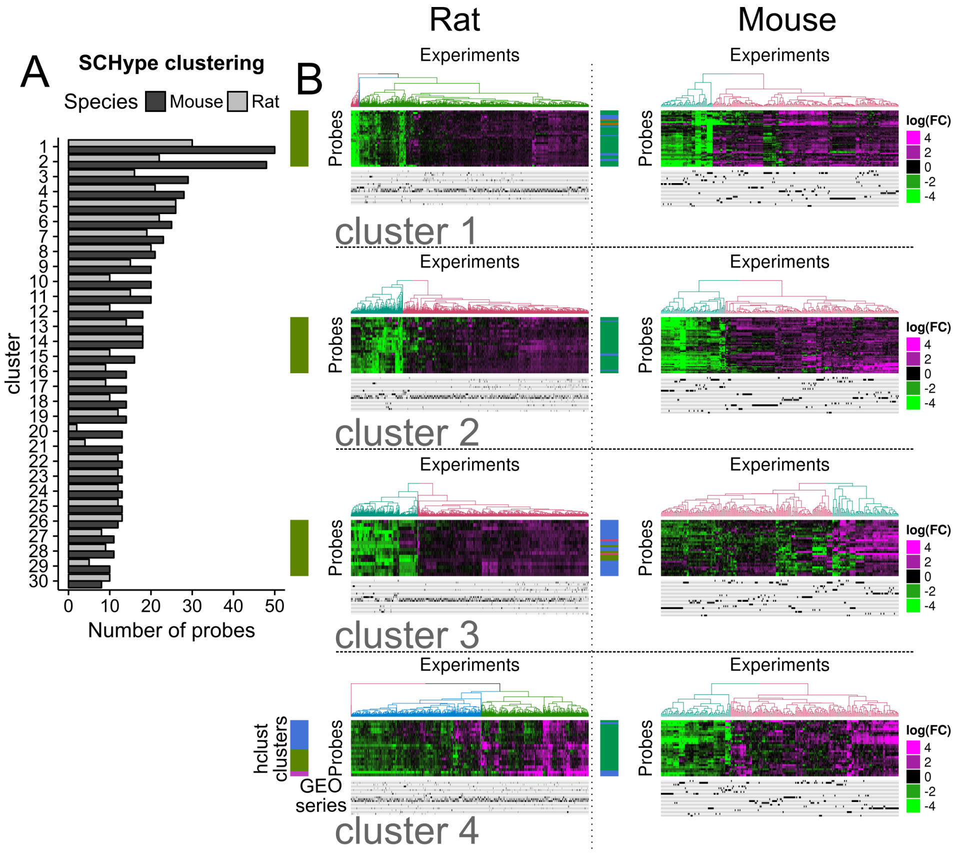

Supplementary figure 6: Co-clustering of rat (middle) and mouse (right) heart data using SCHype. A. Number of mouse (dark grey) and rat (light grey) probes for the heart SCHype clusters with more than 10 probes for each species. X-axis: Number of probes included in each cluster. Y-axis: SCHype predicted cluster ID, numbered according to their number of probes per cluster in decreasing order. B. The biggest four heart SCHype clusters are shown. Genes in mouse and rat in each cluster are homologous to each other. The results of hierarchical clustering for each species is shown as a colour bar on the left. Colour-code matches the experiments trees in supplementary figure 5. Under the heatmap, clustering localisation of experiments from each series are shown in black, one line per series.
